# The Validation of Antibodies Suitable for Flow Cytometric Analysis and Immunopeptidomics of Peptide–MHC Complexes in the Outbred Swiss Albino Mouse Strain

**DOI:** 10.3390/mps8030043

**Published:** 2025-04-24

**Authors:** Shanzou Chung, Isambard G. Knox-Johnson, Sarah E. Gazzard, Runqiu Song, Ngoc H. Le, Luise A. Cullen-McEwen, John F. Bertram, Anthony W. Purcell, Asolina Braun

**Affiliations:** 1 Department of Biochemistry and Molecular Biology, Infection and Immunity Program, Biomedicine Discovery Institute, Monash University, Clayton, VIC 3800, Australia; annie.chung@monash.edu (S.C.); ikno0001@student.monash.edu (I.G.K.-J.); runqiu.song@monash.edu (R.S.); ruby.le@monash.edu (N.H.L.); 2 Department of Anatomy and Developmental Biology, Monash Biomedicine Discovery Institute, Clayton, VIC 3800, Australia; sarah.gazzard1@monash.edu (S.E.G.); luise.cullen-mcewen@monash.edu (L.A.C.-M.); john.bertram@monash.edu (J.F.B.)

**Keywords:** Swiss mice, antigen presentation, immunopeptidomics, major histocompatibility complex, MHC ligands, flow cytometry

## Abstract

Antigen presentation on major histocompatibility complex (MHC) molecules is central to the initiation of immune responses, and a lot of our understanding about the antigen processing and presentation pathway has been gained through studies in mice. MHC molecules are the most genetically diverse genes; consequently, mouse strains differ substantially in their MHC make up and resulting antigen presentation. Swiss mice are commonly used in pharmacological research, yet our understanding of antigen presentation in this strain is surprisingly limited. Here, we have tested a range of anti-MHC antibodies and present a range of clones suitable to analyse MHC class I and class II molecules in Swiss mice who have the H2-q MHC haplotype. Moreover, we demonstrate using immunopeptidomics that clones 28-12-8, 34-1-2, MKD6, and N22 are also suited to isolate MHC class I and class II ligands in this mouse strain. Thus, this work also establishes a first experimental account of the H2-q-derived thymus and spleen immunopeptidome in Swiss mice which bears strong resemblance with ligands isolated from the H2-d MHC haplotype of Balb/C mice. The analysis of source proteins shows common but also organ- and function-specific antigen presentation in line with the involvement of the thymus in tolerance induction and the function of the spleen as a site of immune responses.

## 1. Introduction

Major histocompatibility complex (MHC) molecules play a central role in the immune system by presenting peptides (also known as antigens) to T cells, thereby initiating immune responses. These molecules are essential for the recognition of self and non-self, making them key players in the body’s defence mechanisms against pathogens and cancer, but they also play an important role in the context of autoimmunity and transplant rejection. MHC molecules are polygenic and polymorphic, making them the most diverse genes. Their polygenic nature enhances the ability of an individual’s immune system to recognise a wide array of pathogens. At the same time, the polymorphic diversity at the population level is crucial for the survival of mammalian species, as it increases the likelihood that some individuals within a population will be able to mount an effective immune response against emerging infectious diseases.

Immunopeptidomics is a branch of proteomics that focuses on the study of peptide antigens bound to MHC molecules [1]. By analysing the entire breadth of the antigenic peptide repertoire, immunopeptidomics not only provides insights into the antigen processing and presentation pathway but also aids in the identification of potential targets for vaccine development and immunotherapies. Advances in mass spectrometry and bioinformatics have greatly enhanced the ability to detect and quantify these peptides. However, some limitations still persist due to the extensive diversity of MHC variants, which can complicate the analysis of the immunopeptidome at the population level.

Mice have long been a cornerstone of immunological and MHC research due to the wealth of genetically modified strains and their well-characterised immune systems. Among these, the C57BL/6 and BALB/c strains are the most extensively studied, providing an insight into immune responses and the aetiology of diseases. However, there remains a significant gap in our understanding of MHC antigen presentation in other strains, including Swiss mice that are the focus of this study.

These mice, also known as Swiss Webster and CD-1 mice, are a versatile outbred albino strain. Unlike the inbred C57BL/6 and BALB/c strains, outbred Swiss mice exhibit greater genetic diversity, making them valuable for studies requiring a broader genetic background [2]. This diversity can provide more generalisable results, particularly in toxicology, pharmacology, and general biomedical research. Furthermore, Swiss mice are exceptionally good breeders and used where large litter sizes or embryo transfers are required.

Somewhat surprisingly, despite their widespread use in biomedical research, little is known about the MHC haplotype of Swiss mice, and detailed information on antibody clones that recognise MHC allotypes specifically expressed by Swiss mice is lacking. It must be noted that the MHC haplotypes of C57BL/6, BALB/c, and Swiss mice exhibit significant differences when examined via serological typing, reflecting their distinct genetic backgrounds. C57BL/6 mice possess the H-2b haplotype, characterised by specific MHC class I (H-2Kb, H-2Db) and class II (I-Ab) molecules. This haplotype is associated with a robust immune response and is widely used in immunological research. BALB/c mice carry the H-2d haplotype, with H-2Kd, H-2Dd, and H-2Ld (MHC class I) and I-Ad and I-Ed (MHC class II) molecules. This haplotype is known for its susceptibility to certain infections and tumours, making BALB/c mice valuable in cancer and infectious disease studies. For Swiss mice, it is known that the SWR/J inbred sub-strain carries the H-2q haplotype: MHC class I H-2Kq and H-2Dq molecules and class II I-Aq molecules. Some limited information exists on selected antibody clones recognising the H-2q haplotype for applications like flow cytometry; however, to date, no information is available regarding the antigen peptide repertoire (immunopeptidome) presented by H-2q MHC molecules. A further complication is that most data for the H-2q haplotype come from B10 and DBA/1 mice, not Swiss mice. Accordingly, widely used immunopeptidomics tools like NetMHC and the MHC Motif Atlas mostly do not provide an opportunity to choose MHC allotypes from the H-2q haplotype [3,4]. This gap in knowledge limits the full potential of Swiss mice as a genetically diverse model organism in immunological research. By addressing it, we aimed to enhance the utility of Swiss mice, thereby expanding our understanding of strain-specific murine MHC diversity and providing insights into antigen presentation in this strain.

## 2. Materials and Methods

### 2.1. Mice

Swiss mice were imported into Monash University from the Australian CSL laboratories prior to 1997 and maintained as an isolated colony at the Animal Services Monash University (Asmu) for >28 years. The colony is referred to here as Asmu:Swiss or Swiss mice. A rotational Poiley breeding system was applied throughout to maintain genetic diversity in this outbred population. Further historic information about Australian-bred Swiss lines and their origins has been covered by Cui et al. [5]. To analyse thymus pre-involution, organs of 20-day-old mice were collected for analysis. (Flow cytometry and immunopeptidomics of adult mice yielded similar results). SNP genotyping was performed by Transnetyx/Neogen using the MiniMUGA assay v2.3.13 [6].

### 2.2. MHC Cell Surface Staining

Swiss mouse spleens were processed into single-cell suspensions, cryopreserved in 90% heat-inactivated foetal calf serum (Gibco)/10% dimethyl sulfoxide (Sigma-Aldrich, St. Louis, MO, USA), and stored in liquid nitrogen until further use. For flow cytometry, splenocytes were thawed, resuspended, washed twice in phosphate-buffered saline (PBS), and subsequently stained for MHC surface expression. The CT26 (BalbC), DC2.4 (C57BL/6), and 9004 (human) cell lines were used as controls. All incubation steps were performed in the dark on ice, with washing steps carried out by centrifuging the plate at 445× *g* between staining steps using FACS buffer (2% foetal calf serum and 2.5 mM ethylenediaminetetraacetic acid in PBS). Briefly, 3 × 10^5^ cells were plated onto 96-well plates. Splenocytes and control cell lines were first incubated with anti-CD16/CD32 Fc block in cold FACS buffer for 15 min, followed by the addition of primary antibodies (refer to Table 1) for 30 min. MHC-specific antibodies used in this experiment were produced and purified in-house except for M5-114-15-2. Afterwards, cells were washed and incubated with the appropriate secondary antibody (APC or FITC) or FACS buffer for 30 min, followed by another wash. Splenocytes were then stained with CD4 PeCy7, while control cell lines were resuspended in FACS buffer for 15 min. Finally, cells were washed again, resuspended in FACS buffer containing 0.1 µg/mL DAPI, and acquired through flow cytometry using the LSR Fortessa X-20 flow cytometer (BD Biosciences, Franklin Lakes, NJ, USA). The data were analysed using FlowJo software, version 10.9.0 (BD Biosciences).

### 2.3. Small Scale Immunoaffinity Purification

To prepare tissue lysates, 400 µL of a lysis buffer [0.5% IGEPAL (Sigma Aldrich), 50 mM Tris (pH 8.0, Invitrogen, Thermo Fisher Scientific, Waltham, MA, USA), 150 mM NaCl (Supelco), and one-quarter equivalent of a cOmplete™ Protease Inhibitor Cocktail (Roche)] tablet (dissolved in Optima Water, Fisher Chemical, Thermo Fisher Scientific, Waltham, MA, USA) was added to snap-frozen thymus and spleen tissues that were harvested from Swiss mice and stored at −80 °C. Tissue disruption was performed using a bead mill (TissueLyser LT, Qiagen, Venlo, The Netherlands) at 50 Hz for 2 min. Following homogenization, the lysates were incubated on a tube roller mixer (Ratek, Boronia, Australia) at 4 °C for 45 min to facilitate complete lysis and then centrifuged at 21,000× *g* for 15 min at 4 °C. The clarified lysates were transferred to pre-column tubes containing either protein A agarose resin (CaptivA^®^, Repligen, Waltham, MA, USA) or protein G agarose resin (pH Scientific). The selection of protein resin was based on the antibody isotype and its known binding affinity to protein A or G. The mixtures were incubated with gentle rolling for 1 h at 4 °C to remove non-specific binders. After the removal of the agarose resin, the lysate was transferred onto the immunoaffinity resin. Immunoaffinity resins were prepared by incubating 100 µL of protein A resin each with 200 µg of the antibodies 28-14-8 (targeting H-2Db, H-2Ld, H-2Dq, and H-2Lq), 34-1-2 (H-2Kd, H-2Dd, H-2Kq, and H-2Dq), and MK-D6 (I-Ad and I-Aq) or 100 µL of protein G resin with 200 µg of the N22 antibody (targeting I-A and I-E) in 2 mL Eppendorf protein LoBind tubes for 1 hr. Unbound antibodies were subsequently removed by washing with 10 mL of 1× PBS twice, using a Poly-Prep^®^ Chromatography Column (Bio-Rad, San Francisco, CA, USA), and aliquoted into Eppendorf LoBind tubes in preparation for immunoprecipitation. Peptide–MHC complexes were captured by rolling one pre-cleared organ lysate with each respective antibody resin (one antibody per lysate) overnight at 4 °C. Following the incubation, the peptide–MHC-containing affinity resin was loaded onto pre-washed (three times 10% acetic acid, three times with 1× PBS) Mobicol spin columns (MoBiTec GmbH, Göttingen, Germany) inserted with a 10 µm pore size filter. The immunoaffinity resin was washed three times with 250 mM NaCl in Optima Water, followed by three washes with 1× PBS. The washing steps were performed with a benchtop mini-centrifuge at 1700× *g* to remove detergent and salts. Bound peptide–MHC complexes were eluted in two lots of 200 µL of 10% acetic acid. To liberate the peptides from the MHC and antibodies, the eluate was heated to 70 °C for 10 min using a heat block (Benchmark Scientific isoBlock™) and allowed to cool before being passed through a pre-conditioned (2× wash with 10% acetic acid) 5 kDa centrifugal filter unit (Ultrafree^®^-MC-PLHCC, Merck Millipore, Burlington, MA, USA). The filter units were centrifuged at 16,000× *g* for 45 min, and the peptide eluate flow-through was collected. To recover any residual peptides retained on the filter, an additional 50 µL of 10% acetic acid was added, and centrifugation was repeated. The filtered peptide eluates were then concentrated using a vacuum centrifugation system (Labconco) and stored at −80 °C until further use. Prior to analysis, the concentrated peptide samples were reconstituted in 2% (*v*/*v*) acetonitrile (Fisher Chemical) and 0.1% (*v*/*v*) formic acid (Thermo Fisher Scientific) in Optima Water with a mixture of 11 iRT peptides (Biognosys) spiked in to aid retention time alignment [7]. The peptide samples were sonicated for 10 min, centrifuged at 21,000× *g*, and loaded onto the EvoSep liquid chromatography system to be analysed by Bruker timsTOF Pro2 (Bruker Daltonics, Billerica, MA, USA).

### 2.4. Mass Spectrometry

A hybrid trapped ion mobility-quadrupole time of flight mass spectrometer (Bruker timsTOF Pro 2, Bruker Daltonics) coupled to an EvoSep liquid chromatography system together with a Zoom Whisper 20 SPD method was used for sample analysis. An IonOpticks Aurora Elite column (15 cm × 75um × 1.7 um, with a pore size of 120 A) was used to load HLA ligands. Data-dependent acquisition settings were as follows: *m*/*z* range: 100–1700 mz, capillary voltage:1600 V, target intensity of 30,000, and TIMS ramp of 0.60 to 1.60 Vs/cm^2^ for 166 ms. 

### 2.5. LC-MS/MS Data Analysis

Due to a lack of a specific Swiss mouse proteome, LC-MS/MS data was searched using PEAKS Online 11 (Bioinformatics Solutions Inc., Waterloo, ON, Canada) against the reference Mus musculus proteome 10090 (Uniprot/Swissprot v2023) and a contaminant database containing iRT reference peptides plus the protein A sequence. Peptide identities were subject to strict bioinformatic criteria including the use of a decoy database to calculate the peptide false discovery rate (FDR) of 5%. The following search parameters were used: peptide lengths of 6–30, no enzyme digestion (considers all peptide bond cleavages), no cysteine alkylation, and TimsTOF Pro 2 instrument-specific settings (parent and fragment ion tolerance of 20 ppm and 0.02 Da, respectively). Variable modifications were set to the oxidation of M, the Acetylation N-term, and the deamidation of NQ. Upon sample acquisition, a Peaks DB search was performed followed by a Peaks PTM search a with all default built-in modifications with the same mass tolerance settings as Peaks DB. Immunolyser was used for the visualisation of results [8]. Reference motifs were extracted from https://services.healthtech.dtu.dk/services/NetMHC-4.0/ and https://services.healthtech.dtu.dk/services/NetMHCIIpan-4.0/ accessed on 20 February 2025 [3,9].

## 3. Results

### 3.1. SNP Genotyping of Swiss Mice

**Figure 1:**
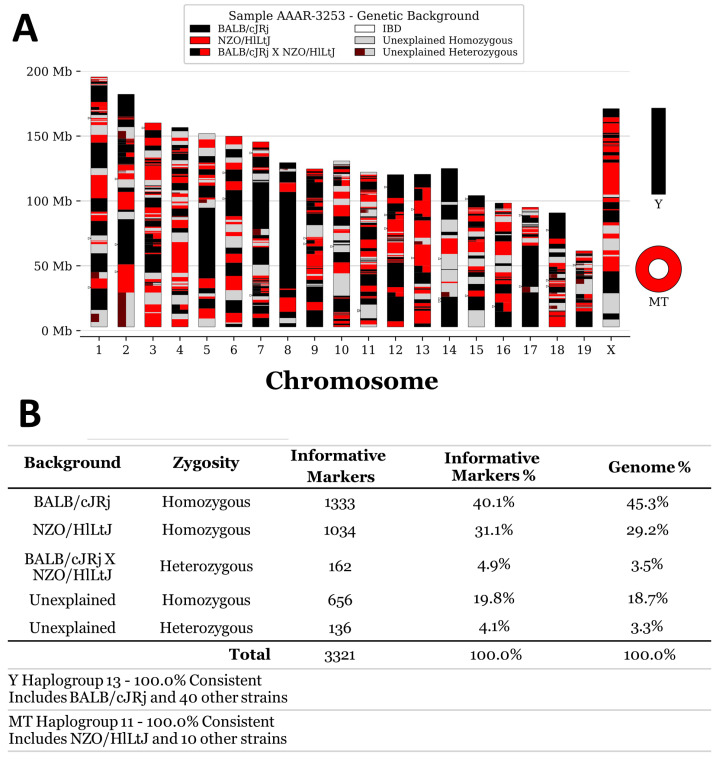
Single nucleotide polymorphism analysis of the Swiss:Asmu strain. MiniMUGA background analysis v2.3.1 was performed on three non-sibling mice; a representative result is shown. (**A**) Chromosomal distribution of 3321 analysed genetic background markers. MT: mitochondrial DNA. (**B**) Tabular summary of the results.

In order to establish the genetic background of the Swiss:Asmu colony held at our facility, we performed SNP genotyping on three mice from this strain using the published MiniMUGA genotyping array [6]. The results confirmed that the strain was outbred. Overall, 42–46% of the genome was consistent with the BALB/cJRj background; 29–32% of the genome was most consistent with either the NZW/LacJ or NZO/HlLtJ background; 3–4% of the loci were heterozygous for BalbC × NZW/NZO, while 22% of the genome contained unique genomic material that could not be matched to known strains in the database (Figure 1). Therefore, the genotyping results confirmed that the Swiss:Asmu mouse strain is outbred with a high diversity and numerous genetic variations within the colony.

### 3.2. Identification of Established Anti-MHC Antibody Clones Capable of Cross-Reactivity with Swiss Cells

**Figure 2:**
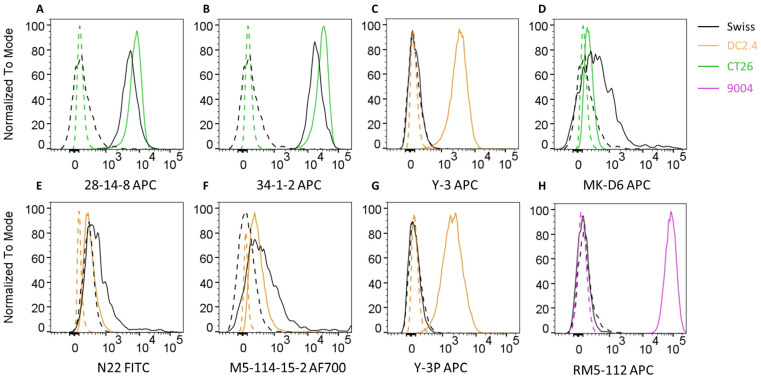
Flow cytometry analysis of MHC surface expression. (**A**–**H**) Histograms depicting MHC expression levels on splenocytes isolated from Swiss mice (Asmu:Swiss, black) and control cell lines, including DC2.4 (C57BL/6, orange), CT26 (BALB/c, green), and 9004 (human, pink). Cells were stained with various mouse MHC-specific antibodies. Dashed lines represent unstained or secondary antibody controls as appropriate, while solid lines indicate MHC-stained populations. APC-conjugated secondary antibody controls were used for panels (**A**–**D**,**G**,**H**); the FITC-conjugated secondary control was used for panel E; the antibody in panel F was directly labelled with Alexa Fluor 700. Gating can be found in Figure S1. Cell lines were pre-gated on live single cells. Swiss splenocytes were pre-gated on live, single, CD4+ splenocytes to avoid any potential non-specific staining on B cells which have Fc receptors. Each histogram represents data from a minimum of two independent experiments.

In order to identify antibodies able to detect MHC class I and MHC class II molecules in Swiss mice, we performed flow cytometric staining of Swiss mouse splenocytes with a range of antibodies known to detect MHC allotypes across a range of different strains of mice (Figure 2, gating in Figure S1). First we tested the 28-14-8 antibody (also known as 28-14-8.S) which binds to the α3 domain of H-2Db and has also been reported to cross-react with H-2Ld, H-2Dq, and H-2Lq but not H-2Kd or H-2Dd [10]. The data on its reactivity with H-2q genes were previously reported based on the staining of cells from B10.AKM, B10.MBR, and DBA/1 mice. We found that the antibody was indeed also able to stain Swiss splenocytes (Figure 2A). Next, we analysed the 34-1-2 antibody (also known as 34-1-2.S) which binds H-2Kd and H-2Dd. It also cross-reacts with other MHC class I allotypes such as H-2Kb/s/r/p/q, and its cross-reactivity to H-2q genes was originally determined based on its reactivity with B10.A mice [11]. We could confirm that the 34-1-2 antibody was able to stain Swiss splenocytes (Figure 2B). In addition, we tested the highly H-2Kb-specific antibody Y-3 which does not cross-react with H-2Kd; Y-3 did not produce a staining on Swiss cells (Figure 2C).

After establishing that two anti-MHC I antibodies, 28-12-8 and 34-1-2, can be used to detect MHC I in Swiss mice, we moved on to test anti-MHC II antibodies. The MK-D6 antibody was raised against B10.D2 splenocytes as an immunogen; it specifically recognises I-Ad and does not react with I-Ak, I-Ab, I-As, I-Af, or I-Aa [12]. In this study, we could show that it does react with the I-Aq of Swiss mice (Figure 2D). We also tested the N22 antibody which is known to interact with a monomorphic part of I-A and I-E molecules [13]. We found that Swiss splenocytes could be successfully stained with N22 (Figure 2E). Next, the M5/114.15.2 antibody was tested. It reacts with a polymorphic epitope on I-Ab, I-Ad, I-Aq, I-Ed, and I-Ek but not I-Af, I-Ak, or I-As [14,15]. Swiss cells stained with M5/114.15.2. Other anti-MHC-II antibodies such as Y-3P (reacts with I-Ab/f/p/q/r/s/u/v haplotypes and weakly with I-Ak as well as rat I-A like molecules) and RM5-112 (pan-HLA class II β-chain) did not produce a staining on Swiss splenocytes (Figure 2F–H) [16]. 

Based on our flow cytometric analysis, we progressed with the antibodies 28-14-8 and 34-1-2 for MHC class I capture as well as MK-D6 and N22 for MHC class II affinity purification in the following experiments.

### 3.3. Immunopeptidomic Analysis in Swiss Mice

**Figure 3:**
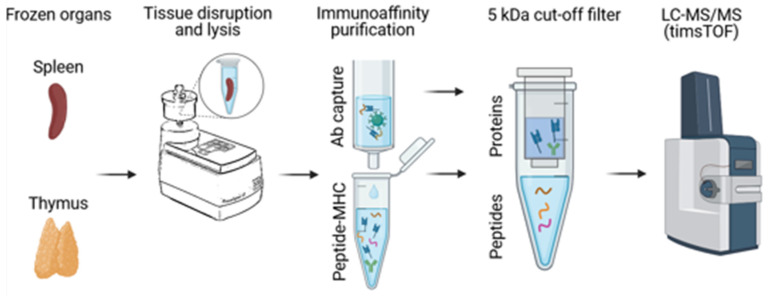
Immunopeptidomics workflow. Frozen Swiss mouse tissues were submersed in lysis buffer and processed into a homogenate using TissueLyser. After 45 min of lysis at 4°C, peptide–MHC complexes were captured using selected antibodies, washed, and eluted using 10% acetic acid. MHC heavy chains, β2m, and antibodies were separated from peptides using 5 kDa molecular weight cut-off filters prior to the analysis of peptides via liquid chromatography–tandem mass spectrometry. Created with Biorender.

Next, we wanted to assess the characteristics of MHC-bound peptides in Swiss mice via immunopeptidomics affinity purification experiments. The workflow was similar to previously published detailed protocols from our lab but included a tissue disruption step in the cell lysis buffer (Figure 3) [1,17]. In brief, frozen organs were added to 400 µL of cold lysis buffer and dissociated at 50 Hz for 2 min in Tissuelyser LT. The lysate was rolled at 4 °C for 45 min; nuclei were spun down, and the lysate was co-incubated with anti-MHC antibodies bound to the protein A/G resin. Peptide–MHC complexes were eluted with 10% acetic acid, and peptides were isolated using 5 kDa molecular weight cut-off filters prior to mass spectrometric analysis.

**Figure 4:**
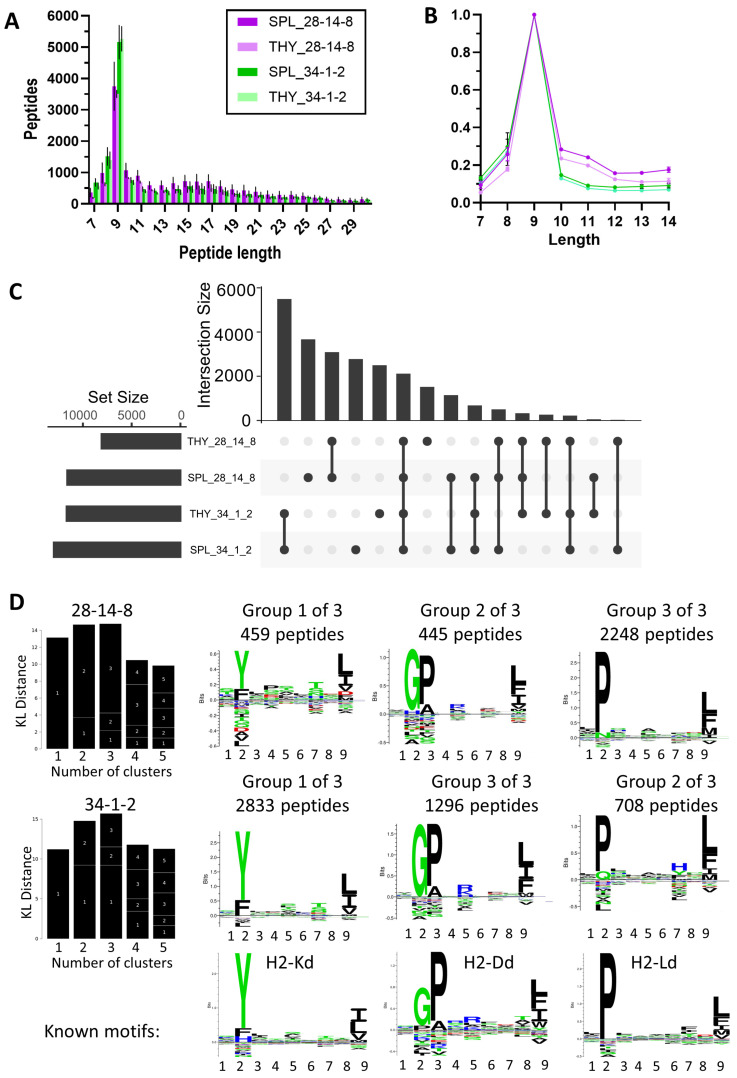
Isolation of MHC I ligands using immunopeptidomics. Antibodies 28-14-8 and 34-1-2 were used to isolate MHC I immunopeptides from the thymus (THY) and spleen (SPL). The analysis was performed using PEAKS Online with a 5% FDR cut-off followed by Immunolyser analysis [6]. (**A**) Mean length distribution and (**B**) normalised length distribution ± SD from three independent experiments are shown. (**C**) Upset plot of common and unique peptides from the combined dataset containing peptides from all replicates is shown. (**D**) Gibbs clustering of 9-mers from representative replicates is shown including the best group number fit based on the Kullbach Leibler (KL) distance. Published NetMHC motifs are shown for reference.

Peptides eluted with the anti-MHC I antibodies 28-12-8 and 34-1-2 in both cases showed a preference for 9-mers as is expected for MHC I-bound peptides (Figure 4A); however, peptides eluted with 28-14-8 contained a higher proportion of longer peptides (Figure 4B). Overlap analysis revealed that there was a high proportion of antibody-specific captured MHC ligands, as well as a significant number of shared peptides (Figure 4C). To further explore the characteristics of the isolated peptides, we performed Gibbs cluster analysis which allows us to identify subsets of peptides contained within a dataset. For both antibodies, a division into three clusters was the best fit, and both antibodies pulled down the same three clusters of peptides (Figure 4D). When comparing the cluster motifs with published and deposited immunopeptidome data from other mouse strains, they showed a close match to H-2Kd, H-2Dd, and H-2Ld (Figure 4D, bottom panel). It needs to be noted that 28-14-8 preferentially pulls down peptides in line with the H2-L allotype, while 34-1-2 preferentially captures peptides that align mostly with the H2-K motif and a significant proportion of peptides with the H2-D motif.

**Figure 5:**
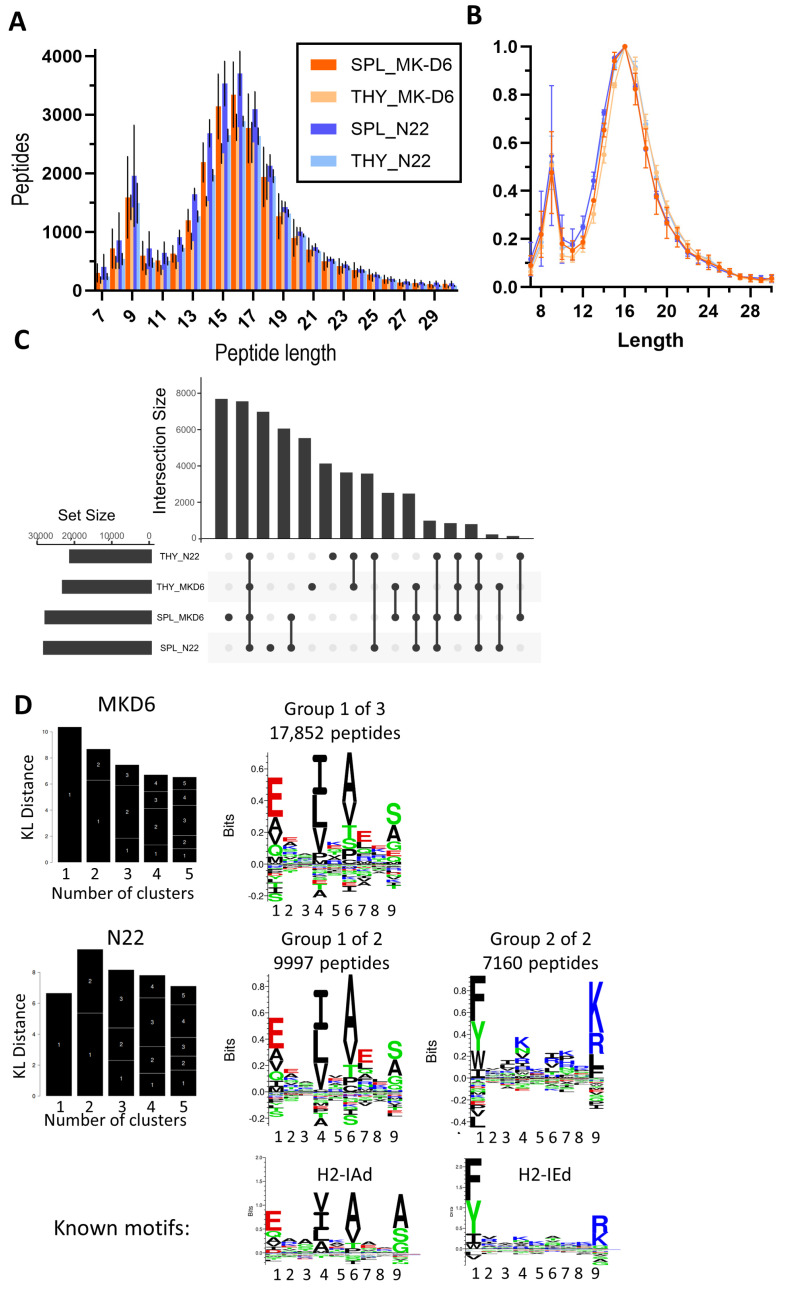
Isolation of MHC II ligands using immunopeptidomics. Antibodies MKD6 and N22 were used to isolate MHC II immunopeptides from the thymus (THY) and spleen (SPL). The analysis was performed using PEAKS Online with a 5% FDR cut-off followed by Immunolyser analysis [6]. (**A**) Mean length distribution and (**B**) normalised length distribution ± SD from three independent experiments are shown. (**C**) Upset plot of common and unique 12–25 mer peptides from the combined dataset containing peptides from all replicates is shown. (**D**) Gibbs clustering of 9-mer cores from representative replicates is shown including the best group number fit based on the Kullbach Leibler (KL) distance. Published NetMHC motifs are shown for reference.

In order to isolate the MHC II-bound immunopeptidome, we chose the MK-D6 and N22 antibody clones. Both antibodies predominantly captured longer peptides, as is typical for MHC II-derived peptides, with 16-mers constituting the predominant length (Figure 5A). However, a considerable contamination of shorter MHC I peptides could be observed, resulting in a second peak around 8–10-mers (Figure 5A,B and Figure S2). Therefore, to analyse the peptide overlap between these antibodies, we filtered for 12–25-mers first before generating an Upset plot. There was substantial overlap in peptides captured by both antibodies, and the main groups of unique peptides were attributed to the source organ rather than the antibody type (Figure 5C). Accordingly, Gibbs clustering revealed the same peptide motif for both antibodies, which is in line with the published H2-IAd motif. However, N22 is also capable of capturing a second group of distinct peptides that display a motif in line with H2-IEd (Figure 5D). 

### 3.4. Comparison of the Thymus- and Spleen-Derived Immunopeptidomes

The two tissues analysed represent immune-relevant organs that fulfil different functions. The spleen is a secondary lymphoid organ involved in the initiation and orchestration of immune responses. The thymus is a primary immune organ specialised in the development and selection of naïve T cells, ensuring that T cells and their T cell receptors recognise antigens presented on MHC molecules (positive selection) while avoiding self-reactivity through the process of negative selection. Expression of autoimmune regulator gene (AIRE) and family zinc finger protein 2 (FEZF2) enables medullary thymic epithelial cells to present tissue-specific antigens to immature T cells, leading to the negative selection of T cells that would recognise tissue-specific antigens [18,19].

To gain an overview of antigen presentation in these two organs, we evaluated the source proteins as captured across different antibodies throughout this project. Thus, the source proteins for the spleen and thymus immunopeptidomes were categorised as shared (74%) and tissue-specific (26%) MHC ligands. Among the unique proportion of proteins, 3578 source proteins were exclusively found in the spleen immunopeptidome, and 3458 exclusive source proteins were sampled within the immunopeptidome of the thymus (Supplementary Data S1). Next, by clustering highly similar proteins into protein groups, these numbers were reduced to 1799 and 1709 unique source protein groups in the spleen and thymus, respectively. We then isolated the top 10 protein groups represented by the highest number of peptide spectral counts (Table 2). In the spleen, the top 10 unique source protein groups were representative of the abundant immune cell populations. For example, complement receptors, immunoglobulins, Fc receptors, and the kinase SYK are all highly expressed in B cells, the main cell type in the spleen (45–50%). By comparison, the top 10 protein groups in the thymus were diverse and bore evidence of numerous tissue-specific antigens, with beta-casein being the most prominent and well-described example.

## 4. Discussion

This study provides information on antibodies able to recognise and isolate various MHC class I and class II alleles in Swiss mice. We identified that the anti-MHC I antibodies 28-14-8 and 34-1-2 both capture all three major class I alleles, yet together they are rather complementary. While 34-1-2 preferably binds to and captures H2-K- and H2D-derived peptides, 28-14-8 is predominantly detecting H2-L alleles. Both antibodies are highly specific to class I MHC molecules. However, for the anti-MHC II antibodies MKD6 and N22, substantial amounts of shorter MHC I peptides which contain H-2K, H-2D, and H-2L motifs in similar proportions are co-isolated. For MHC II immunopeptidome capture, it could therefore be recommended to design elution experiments in a tandem setup by first depleting MHC class I peptides using a mix of 28-14-8 and 34-1-2 followed by the incubation of the remaining cell lysate with anti-MHC class II antibodies. MKD6 can be used when exclusive capture of H2-IA is sought, while N22 is a preferred choice if both H2-IA and H2-IE need to be isolated.

The motif analysis of all antibodies revealed that the H2-q haplotype of Swiss:Asmu mice is similar to the H2-d haplotype of BalbC mice. To date, only very limited genomic sequencing data exist for the Swiss strain, and the only available MHC I protein sequence from the H2-q haplotype is the H2-Kq protein accession (P14428.1) derived from Swiss SWR mice. It displays 98% homology to the H2-Kd protein accession (P01902.1) derived from BalbC/J mice [20,21]. The high similarities in the presented peptides by the H2-q and H2-d haplotypes are also in line with the previously reported cross-reactivity of some antibodies between these two haplotype strains that we could confirm in our flow cytometric analysis. Furthermore, it is in line with the genomic miniMUGA analysis which found that the MHC gene-containing region on chromosome 17 aligns best with the genomic background of the Balb/cJRj strain.

A comparative analysis of the thymus and spleen immunopeptidomes showed a substantial overlap but also divergence between source protein groups in the thymus and spleen, providing insights into the distinct and overlapping functional landscapes of these immune-relevant organs. The presence of shared antigens likely reflects common mechanisms and cell types in these immune organs, while tissue-specific antigens illuminate the unique roles of each organ in shaping immune responses. The data underscore the functional specialisation of the thymus and spleen while also highlighting their complementary roles in displaying common antigens. Future studies could further elucidate the functional implications of these findings by exploring the dynamics of antigen presentation under physiological and pathological conditions.

Overall, this study aids in developing a better understanding of MHC diversity in mice across different immune organs and helps to shed light on antigen presentation in the Swiss mouse strain that is used across different areas of medical research.

## Figures and Tables

**Table 1:** List of antibodies used for flow cytometry.

Antibody	Species Origin/Isotype	Source	Catalogue Number	Amount Used/Dilution Factor
28-14-8	Mouse/IgG2a	In-house	HB-27	2 μg
34-1-2	Mouse/IgG2a	In-house	HB-79	2 μg
MK-D6	Mouse/IgG2a	In-house	HB-3	2 μg
N22	Hamster/IgG	In-house	HB-225	2 μg
RM5-112	Mouse/IgG2a	In-house	N/A	2 μg
Y-3	Mouse/IgG2a	In-house	HB-176	2 μg
Y-3P	Mouse/IgG2a	In-house	HB-183	2 μg
M5-114-15-2 Alexa Fluor 700	Rat/IgG2b	eBioscience	56-5321-82	0.04 μg
CD4 PeCy7	Mouse/IgG2a	BD Biosciences	348799	1:500 dilution
CD16/CD32	Rat/IgG2b	BD Biosciences	553142	1:200 dilution
APC ((F(ab’)2-Goat anti-Mouse IgG)	Goat/IgG	eBioscience	17-4010-82	1:1000 dilution
FITC (Goat anti-hamster (Armenian) IgG)	Goat/polyclonal IgG	BioLegend	405502	1:200 dilution

**Table 2:** Top source protein groups in the thymus and spleen.

Thymus—Top 10 Unique Source Proteins	Spleen—Top 10 Unique Source Proteins
Phospholipase D1	Acidic leucine-rich nuclear phosphoprotein 32 family member E
Major urinary protein (various)	Complement receptor type 2
Keratin, type II cytoskeletal	Ig kappa chain V-III region
Protein TBATA (Thymus, Brain And Testes Associated)	Paired box protein Pax-5
Thymus-specific serine protease	Trem-like transcript 2 protein
Beta-casein	Alpha-synuclein
Epithelial splicing regulatory protein 1	Fc receptor-like protein 1
Proteoglycan 4	40S ribosomal protein S25
Desmoglein-2	Acidic leucine-rich nuclear phosphoprotein 32 family member A
Pro-adrenomedullin	Tyrosine-protein kinase SYK

## Data Availability

Mass spectrometry raw data has been deposited as MassIVE dataset MSV000097693.

## Supplementary Materials

The following supporting information can be downloaded at: https://www.mdpi.com/article/10.3390/mps8030043/s1.
