# Supplementary material for: The Validation of Antibodies Suitable for Flow Cytometric Analysis and Immunopeptidomics of Peptide–MHC Complexes in the Outbred Swiss Albino Mouse Strain
Source: Methods Protoc. 2025 Apr 24;8(3):43. doi: 10.3390/mps8030043 (PMC12101307; doi:10.3390/mps8030043)
Supplement: Supplementary file 1 [file mps-08-00043-s001.zip › mps-3522302-supplementary.pdf]

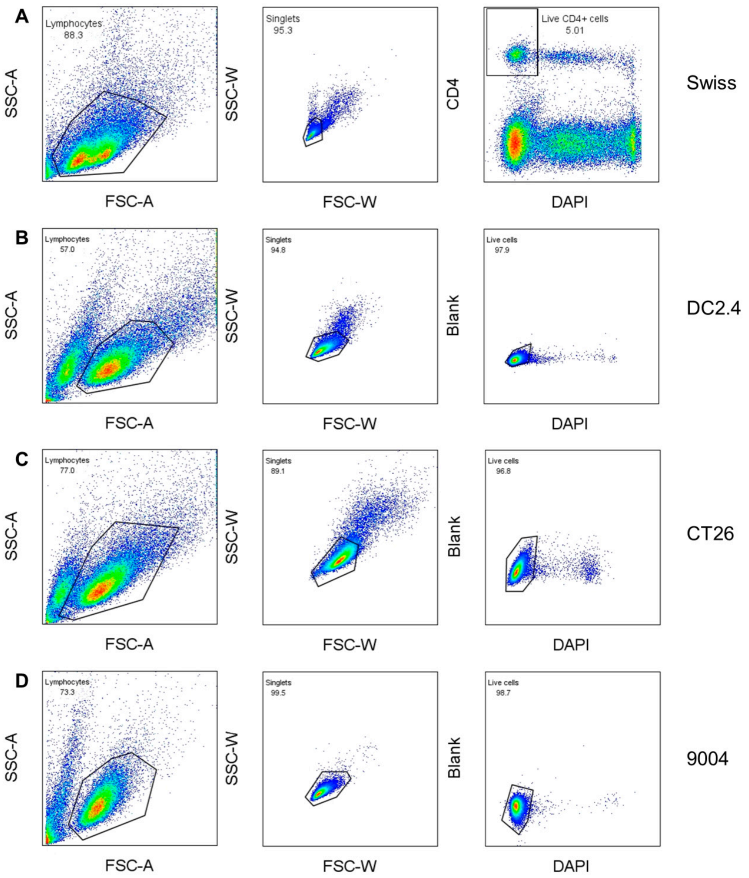

Figure S1. Gating strategy for flow cytometry analysis of MHC surface expression in Swiss splenocytes and control cell lines (DC2.4, CT26 and 9004). (A) Swiss splenocytes were gated based on forward scatter (FSC-A) and side scatter (SSC-A) properties to exclude debris, followed by doublet discrimination (FSC-W, SSC-W). Live $CD4^{+}$ cells were identified using DAPI and CD4 staining. (B-D) DC2.4, CT26 and 9004 cell lines were gated on FSC, SSC, with dead cells excluded based on DAPI staining.

N22, 9-mers

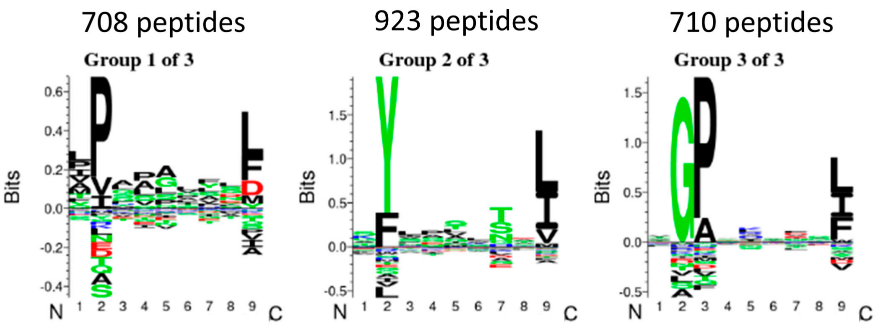

MKD6, 9-mers

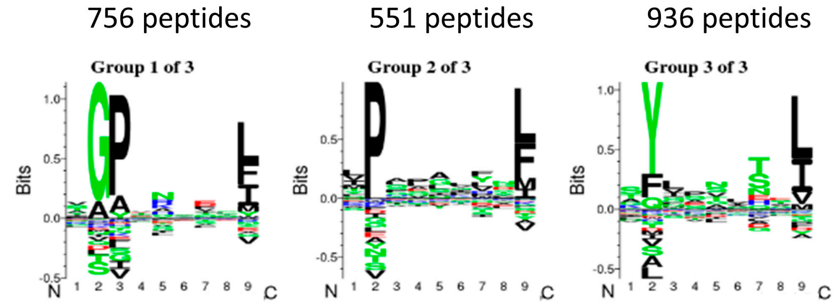

Figure S2. Analysis of 9-mers in MHC II elutions
Antibodies MKD6 and N22 were used to isolate MHC II immunopeptides from thymus (THY) and spleen (SPL). Analysis was performed using PEAKS Online and a 5% FDR cut-off followed by Immunolyser analysis [6]. Gibbs clustering of 9-mers from representative spleen replicates is shown.
